# Synergistic Effects of Oligochitosan and Pyraclostrobin in Controlling Leaf Spot Disease in *Pseudostellaria heterophylla*

**DOI:** 10.3390/antibiotics13020128

**Published:** 2024-01-27

**Authors:** Cheng Zhang, Chenglin Tang, Qiuping Wang, Yue Su, Qinghai Zhang

**Affiliations:** 1 Key Laboratory of Environmental Pollution Monitoring and Disease of Ministry of Education, School of Public Health, Guizhou Medical University, Guiyang 550025, China; chengz76@gmc.edu.cn; 2 Guizhou Crop Technology Extension Station, Agriculture and Rural Affairs Department of Guizhou Province, Guiyang 550001, China; cl_tang520@163.com; 3 Department of Food and Medicine, Guizhou Vocational College of Agriculture, Qingzhen 551400, China; qpwang518@aliyun.com; 4 State Key Laboratory of Environmental Geochemistry, Institute of Geochemistry, Chinese Academy of Sciences, Guiyang 550081, China

**Keywords:** pyraclostrobin, oligochitosan, leaf spot disease, *Pseudostellaria heterophylla*

## Abstract

*Pseudostellaria heterophylla* (or Taizishen in Chinese), a medicinal, edible, and ornamental Chinese herb, is seriously affected by leaf spot disease (LSD). Oligochitosan is a natural agricultural antibiotic that is produced via the degradation of chitosan, which is deacetylated from chitin; pyraclostrobin is a broad-spectrum and efficient strobilurin fungicide. In this work, the ability of pyraclostrobin, oligochitosan, and their formula to manage *P. heterophylla* leaf spot disease and their role in its resistance, leaf photosynthesis, agronomic plant traits, root growth, and root quality were studied. The results show that the joint application of oligochitosan and low-dosage pyraclostrobin could control LSD more efficiently, with control effects of 85.75–87.49% compared to high-dosage pyraclostrobin or oligochitosan alone. Concurrently, the application of this formula could more effectively improve the resistance, leaf photosynthesis, agronomic plant traits, root yield, and medicinal quality of *P. heterophylla*, as well as reduce the application of pyraclostrobin. This finding suggests that 30% pyraclostrobin suspension concentrate (SC) 1500-time + 5% oligosaccharin aqueous solutions (AS) 500-time diluent can be recommended for use as a feasible formula to manage LSD and reduce the application of chemical pesticides.

## 1. Introduction

*Pseudostellaria heterophylla* (Taizishen in Chinese), a medicinal, edible, and ornamental plant of Caryophyllaceae that is rich in polysaccharides, flavonoids, saponins, minerals, and amino acids, has great medicinal, nutritional, and economic value [1,2,3,4]. Its dried tuberous root has many beneficial and pharmacological functions; it is able to inhibit tumor cells, moisten the lungs, protect the myocardium, invigorate the spleen and stomach, benefit the blood, and enhance immunity [4,5,6,7]. *P. heterophylla* has long, spindle-shaped roots, opposite leaves, and axillary flowers. It is planted from October to December of the previous year and harvested from July to September of the following year [8]. It is mainly produced in China, far east Russia, the Korean Peninsula, and Japan, where its production vitalizes rural areas and alleviates poverty; it is widely produced in Guizhou Province, China, where there is over 20,000 hm^2^ of planting area [8]. Moreover, LSD is a serious fungal disease that affects *P. heterophylla*, frequently leading to economic losses of more than 50% [9,10,11,12]. In the early stage of LSD, brown round or irregular spots appear on the leaves; these then gradually expand, and the center of the spots becomes grayish white or light yellow-brown before dead spots are formed in the later stage. Plants can also die in severe cases [9,10,11,12]. As a consequence, some fungicides, biomolecules, and microbes are used to control LSD, including tebuconazole, pyraclostrobin, trifloxystrobin, azoxystrobin, difenoconazole, eugenol, and *Bacillus subtilis*, etc. [9,10,11,12,13]. Since LSD seriously constricts the growth of *P. heterophylla*, there is a significant need for multiple, practicable, and alternative control measures to enhance the sustainable development of the industry.

Pyraclostrobin, a broad-spectrum and efficient strobilurin fungicide, is one of the most commonly applied and sold fungicides [14,15]. During mitochondrial respiration in cells, it disrupts the charge transfer between cytochrome c1 and cytochrome b, making the mitochondria unable to properly provide the energy required for cell metabolism, thus taking on a bactericidal role [16,17,18]. Meanwhile, as a systemic, protective, and therapeutic fungicide, pyraclostrobin can protect crops, vegetables, and fruit trees from many fungal pathogens, including *Basidiomycetes*, *Oomycetes*, *Hemimycetes*, and *Ascomycetes*, etc. [19,20,21]. In a previous study, a 60% pyraclostrobin/metiram water-dispersible granule containing 5% pyraclostrobin exhibited good activity against the leaf spot pathogen *Alteraria tenuissima*, with an EC_50_ value of 531.211 μg mL^−1^ [9]. Subsequently, Yang et al. [11] investigated the role of the application of fungicide in the disease control process of *P. heterophylla* in planting enterprises or individual households in Guizhou Province; they found that pyraclostrobin was frequently used to control LSD. However, pyraclostrobin poses a hidden threat to the environment, animals, hydrobios, and humans due to the expansion of its targets and application dosage and area [22,23,24,25]. Moreover, the resistance of pathogens to pyraclostrobin will be easily induced with the increase in its use [26]. Interestingly, our recent study showed that chitosan is an effective adjuvant and can be used to promote pyraclostrobin’s control of the powdery mildew of *Rose roxburghi* and reduce the application of pyraclostrobin [21]. Correspondingly, by seeking natural biomolecules or products, such as the synergists of pyraclostrobin, in order to efficiently prevent LSD in *P. heterophylla*, the application and potential risks posed by pyraclostrobin can be reduced, and pathogen resistance can be effectively ameliorated.

Oligochitosan is a natural agricultural antibiotic that is produced by the degradation of chitosan, which is deacetylated from chitin; it is widely applied due to its ability to regulate the response of plants to infection with pathogens and its ability to enhance the growth of plants in agriculture [27,28]. It is also widely used in food, the chemical and energy industries, environmental protection, medicine, and other fields due to its good water-soluble, film-forming, antibacterial, antioxidant, and renewable characteristics [27,28]. Oligochitosan can induce disease resistance in plants, produce immunity, and kill many fungi, bacteria, and viruses; it is also able to control various diseases such as wheat mosaic disease, rice blast, tomato blight, etc. [28,29]. In addition to inhibiting the growth of some pathogens, it can stimulate the gene expression of disease-resistant substances in plants, including chitinase, glucanase, phytoalexin, and PR protein, and activate cells to enhance the plant’s resistance and growth [27,28,30,31,32]. For instance, in one study, the stem and biomass of tomatoes were obviously enhanced by the foliar application of oligochitosan [33,34]. Moreover, glutathione–oligochitosan had a lower incidence of 0.66~13% against the downy mildew on blackberries than that of the tested fungicides or control [31]. Of note, Wang et al. [35] found that oligochitosan combined with tebuconazole could reliably control soft rot in kiwifruit and notably enhance its resistance substances and enzyme activity; it could also effectively promote the growth and quality of kiwifruit. Nevertheless, there is currently no literature available regarding the process by which oligochitosan controls LSD in *P. heterophylla*. Recently, our group demonstrated that the effects of difenoconazole against leaf spot disease in *P. heterophylla* could be effectively enhanced by chitosan [36]. Thus, whether oligochitosan can promote the effects of pyraclostrobin against LSD and whether their combination could efficiently enhance the growth of *P. heterophylla* are also worth further exploration.

In this study, the ability of pyraclostrobin, oligochitosan, and their formula to control LSD in *P. heterophylla* was first investigated. Simultaneously, its resistance and photosynthesis were determined. Subsequently, the quality of its roots and growth were also evaluated. The aim of this study was to excogitate an alternative, practicable, and environmentally friendly agronomic measure for managing LSD in *P. heterophylla*.

## 2. Results

### 2.1. Effects of Pyraclostrobin and Oligochitosan on LSD

Table 1 displays the ability of pyraclostrobin and oligochitosan and pyraclostrobin and oligochitosan to control LSD. P 1500 + O 500, P 1000, P 1500, O 500, and O 1000 clearly (*p* < 0.01) attenuated the disease index at 15 d and 30 d after the last spraying compared to the control. Oligochitosan exhibited a good potential to control LSD, while pyraclostrobin displayed a good capacity for control. Synergistically, P 1500 + O 500 was able to control LSD well, with effects of 87.49~85.75%; these values are significantly (*p* < 0.05) higher than those of P 1000 and clearly (*p* < 0.01) exceeded those of P 1500, O 500, and O 1000. Moreover, O 500 and O 1000 exhibited a unique phenomenon, that is, their ability to control LSD increased with the extension of the investigation time, indicating that they have favorable persistence and inducibility. These results demonstrate that oligochitosan mixed together with quick-acting and low-dosage pyraclostrobin could more effectively control LSD compared to high-dosage pyraclostrobin; this combination also effectively reduced the pyraclostrobin application dosage.

### 2.2. Effects of Pyraclostrobin and Oligochitosan on Disease Resistance

**Figure 1:**
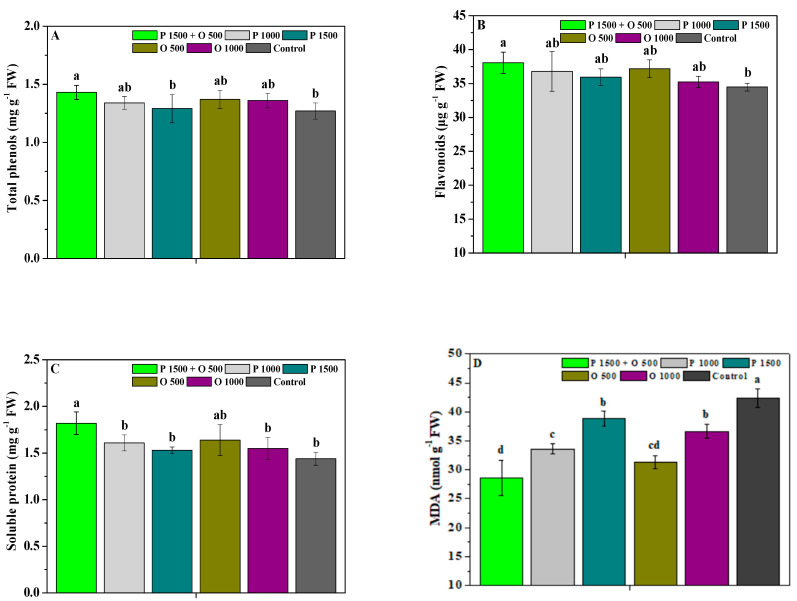
The effects of pyraclostrobin and oligochitosan on the phenols (**A**), flavonoids (**B**), soluble proteins (**C**), and MDA (**D**) of leaves. The error bar indicates the standard deviation; different lowercase letters indicate significant differences at the 5% (*p* < 0.05) level, see below.

The effects of pyraclostrobin and oligochitosan, pyraclostrobin, and oligochitosan on the total phenols, flavonoids, soluble protein, and MDA of *P. heterophylla* leaves are shown in Figure 1. P 1000, P 1500, O 500, and O 1000 slightly enhanced the total phenols, flavonoids, and soluble protein of the leaves compared to the control, and there were no clear differences in these parameters (*p* < 0.05) in the above treatments. Compared to the control, P 1500 + O 500, P 1000, P 1500, O 500, and O 1000 significantly (*p* < 0.05) decreased the MDA content of the leaves. Nevertheless, P 1500 + O 500 significantly (*p* < 0.05) promoted the phenol, flavonoid, and protein contents of the leaves compared to the control. Moreover, the total phenol content of the *P. heterophylla* leaves treated with P 1500 + O 500 was obviously (*p* < 0.05) greater than that of P 1500 and slightly greater than that of P 1000, O 500, and O 1000; its flavonoid content was slightly higher than that of P 1000, P 1500, O 500, and O 1000; its soluble protein content was significantly (*p* < 0.05) higher than that of P 1000, P 1500, and O 1000, and slightly higher than that of O 500; and its MDA content was significantly (*p* < 0.05) lower than that of P 1000, P 1500, O 500, and O 1000. These results show that oligochitosan combined with pyraclostrobin could effectively enhance the promoting or inhibiting roles of pyraclostrobin or oligochitosan in the resistance of *P. heterophylla* leaves; this combination also displayed the favorable potential to improve *P. heterophylla* resistance.

**Figure 2:**
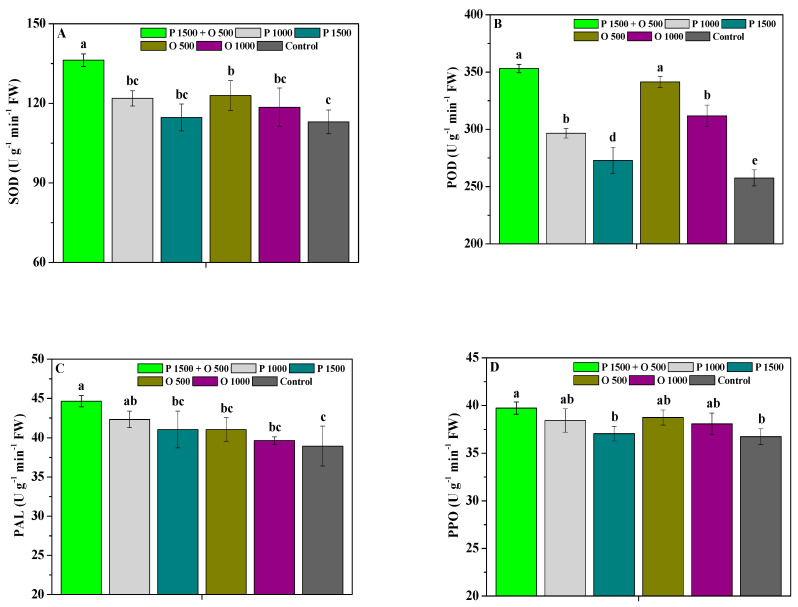
The effects of pyraclostrobin and oligochitosan on the SOD (**A**), POD (**B**), PAL (**C**), and PPO (**D**) activities of leaves; different lowercase letters indicate significant differences at the 5% (*p* < 0.05) level.

The effects of pyraclostrobin and oligochitosan, pyraclostrobin, and oligochitosan on the SOD, POD, PAL, and PPO activities of leaves are depicted in Figure 2. P 1500 + O 500 and O 500 significantly (*p* < 0.05) promoted the SOD activity of *P. heterophylla* leaves; P 1500 + O 500, P 1000, P 1500, O 500, and O 1000 significantly (*p* < 0.05) enhanced their POD activity compared to the control; P 1500 + O 500 and P 1000 significantly (*p* < 0.05) improved their PAL activity; and P 1500 + O 500 significantly (*p* < 0.05) enhanced their PPO activity. Additionally, the SOD activity of the leaves treated with P 1500 + O 500 was significantly (*p* < 0.05) greater than that of those treated with P 1000, P 1500, O 500, and O 1000; their POD activity was significantly (*p* < 0.05) greater than that of those treated with P 1000, P 1500, and O 1000; their PAL activity was significantly (*p* < 0.05) greater than that of those treated with P 1500, O 500, and O 1000; and their PPO activity was significantly (*p* < 0.05) greater than that of those treated with P 1500. These results show that oligochitosan combined with pyraclostrobin clearly promotes the effects of their individual application on the resistance enzyme activities of *P. heterophylla*, and thus effectively enhances the resistance of *P. heterophylla*.

### 2.3. Effects of Pyraclostrobin and Oligochitosan on Photosynthetic Capability

**Figure 3:**
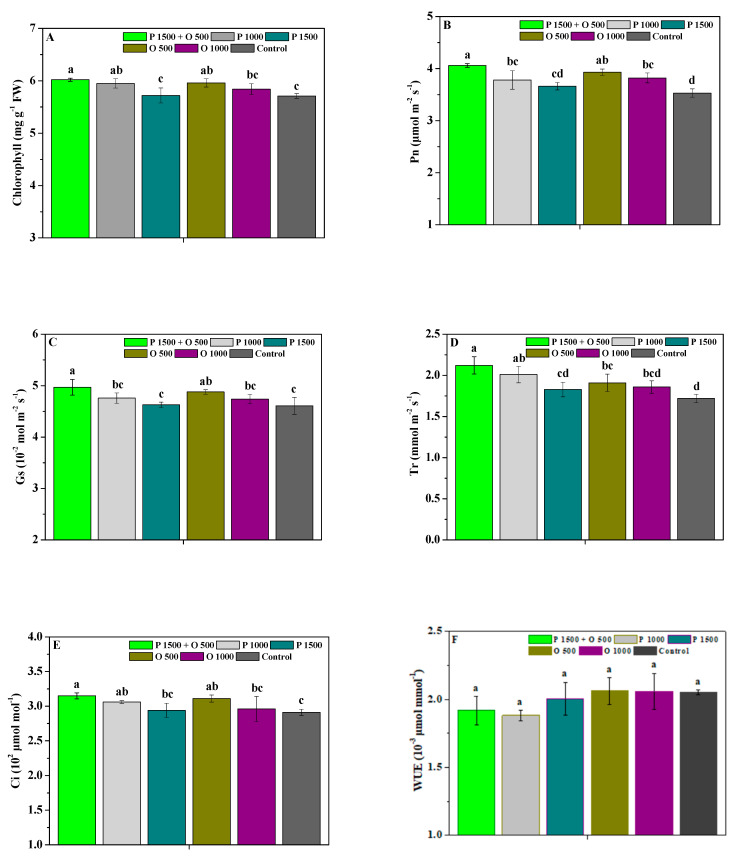
The effects of pyraclostrobin and oligochitosan on the chlorophyll (**A**), Pn (**B**), Gs (**C**), Tr (**D**), Ci (**E**), and WUE (**F**) of leaves; different lowercase letters indicate significant differences at the 5% (*p* < 0.05) level.

Figure 3 shows the effects of pyraclostrobin and oligochitosan, pyraclostrobin, and oligochitosan on the photosynthetic capacity of *P. heterophylla*. P 1500 + O 500, P 1000, and O 500 significantly (*p* < 0.05) promoted the chlorophyll and Ci contents of *P. heterophylla* compared to the control; P 1500 + O 500, P 1000, O 500, and O 1000 significantly (*p* < 0.05) enhanced its Pn content; P 1500 + O 500 and O 500 significantly (*p* < 0.05) improved its Gs content; and P 1500 + O 500, P 1000, and O 500 significantly (*p* < 0.05) enhanced its Tr content. Nevertheless, the chlorophyll, Pn, Gs, Tr, and Ci contents of the leaves treated with P 1500 + O 500 were 1.02, 1.06, 1.01, and 1.03-fold; 1.07, 1.10, 1.03, and 1.05-fold; 1.06, 1.09, 1.03, and 1.06-fold; 1.07, 1.17, 1.13, and 1.16-fold; and 1.04, 1.08, 1.02, and 1.07-fold greater compared to the P 1000, P 1500, O 500, and O 1000 treatments, respectively. These findings show that the joint application of oligochitosan and pyraclostrobin could more effectively enhance the photosynthetic capacity of *P. heterophylla* and reliably enhance its growth.

### 2.4. Effects of Pyraclostrobin and Oligochitosan on Agronomic Trait

**Figure 4:**
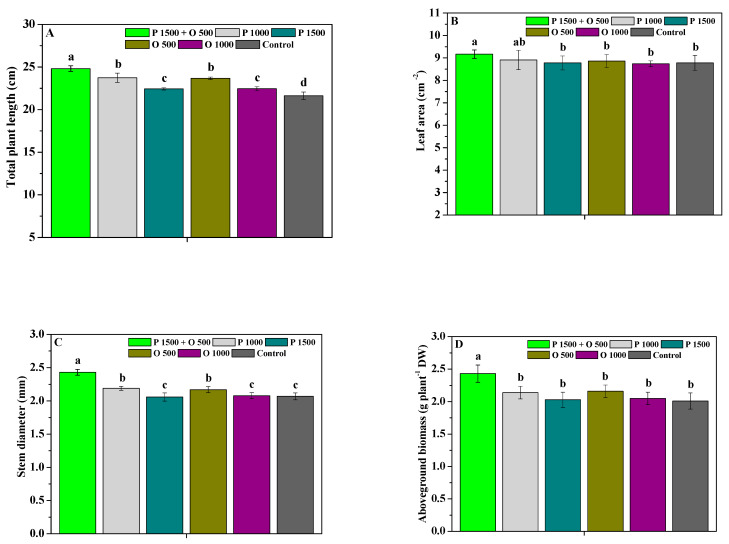

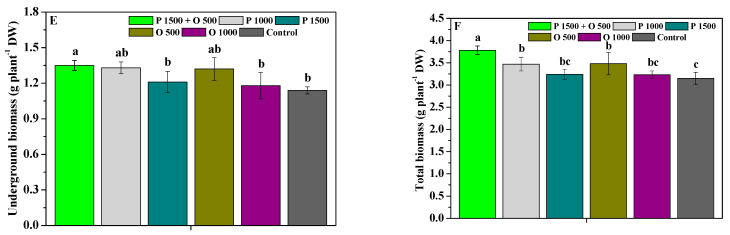
The effects of pyraclostrobin and oligochitosan on the total plant length (**A**), leaf area (**B**), stem diameter (**C**), aboveground biomass (**D**), underground biomass (**E**), and total biomass (**F**) of plants; different lowercase letters indicate significant differences at the 5% (*p* < 0.05) level.

Figure 4 shows the effects of pyraclostrobin and oligochitosan, pyraclostrobin, and oligochitosan on the agronomic traits of *P. heterophylla*. P 1500 + O 500, P 1000, P 1500, O 500, and O 1000 significantly (*p* < 0.05) enhanced the total plant length of *P. heterophylla* compared to the control; P 1500 + O 500, P 1000, and O 500 significantly (*p* < 0.05) increased its stem diameter and total biomass; and P 1500 + O 500 significantly (*p* < 0.05) enhanced its leaf area, aboveground biomass, and underground biomass. Moreover, the total plant length, leaf area, stem diameter, aboveground biomass, underground biomass, and total biomass of the *P. heterophylla* plants treated with P 1500 + O 500 were 1.15, 1.04, 1.17, 1.21, 1.18, and 1.20-fold greater compared to those of the control, respectively. Nevertheless, the ability of P 1500 + O 500 to enhance the agronomic traits of *P. heterophylla* was better than that of P 1000, P 1500, O 500, and O 1000. These findings indicate that the joint application of oligochitosan and pyraclostrobin could more effectively enhance the growth and agronomic traits of *P. heterophylla*.

### 2.5. Effects of Pyraclostrobin and Oligochitosan on Yield and Quality

**Figure 5:**
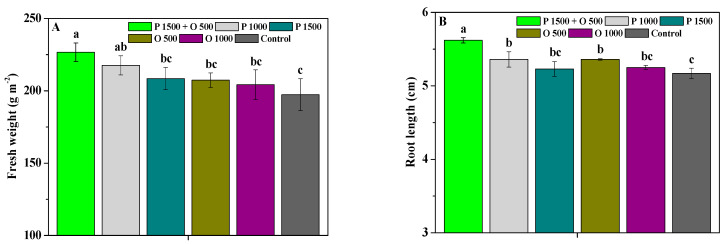

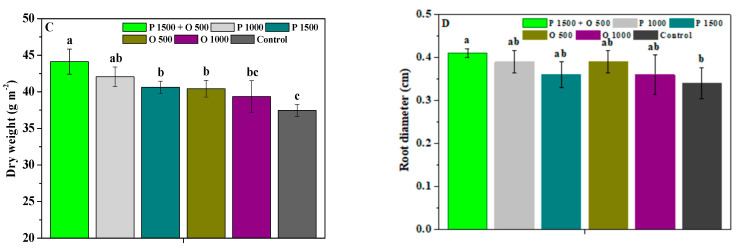
The effects of pyraclostrobin and oligochitosan on the fresh weight (**A**), root length (**B**), dry weight (**C**), and root diameter (**D**) of roots; different lowercase letters indicate significant differences at the 5% (*p* < 0.05) level.

Figure 5 shows the effects of pyraclostrobin and oligochitosan, pyraclostrobin, and oligochitosan on the weight, length, and diameter of *P. heterophylla* roots. Compared to the control, P 1500 + O 500, P 1000, P 1500, O 500, and O 1000 notably enhanced the fresh weight, dry weight, length, and diameter of the roots. P 1500 + O 500 significantly enhanced yield performance of *P. heterophylla*, with a fresh weight, dry weight, length, and diameter of 226.68 g m^−2^, 44.12 g m^−2^, 5.62 cm, and 0.41 cm; these values are 1.05, 1.10, 1.10, 1.12, and 1.16-fold; 1.05, 1.09, 1.09, 1.12, and 1.18-fold; 1.05, 1.08, 1.05, 1.08, and 1.09-fold; and 1.03, 1.11, 1.03, 1.11, and 1.18-fold greater compared to P 1000, P 1500, O 500, O 1000, and the control, respectively. These findings indicate that pyraclostrobin and oligochitosan can promote the root yield of *P. heterophylla* more reliably.

**Figure 6:**
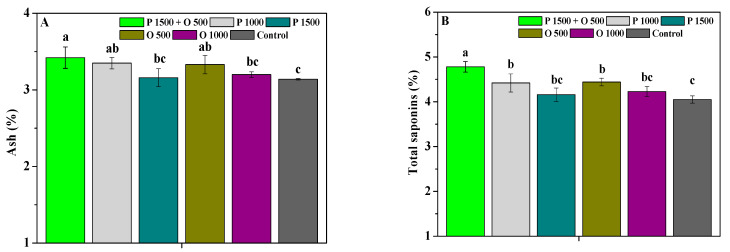

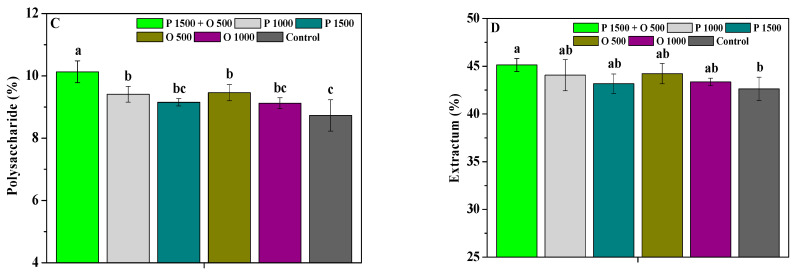
The effects of pyraclostrobin and oligochitosan on ash (**A**), total saponins (**B**), polysaccharide (**C**), and extractum (**D**) of roots; different lowercase letters indicate significant differences at the 5% (*p* < 0.05) level.

The effects of pyraclostrobin and oligochitosan, pyraclostrobin, and oligochitosan on the ash, total saponins, polysaccharide, and extractum contents of the roots are shown in Figure 6. P 1500 + O 500, P 1000, and O 500 significantly (*p* < 0.05) promoted the ash, total saponins, and polysaccharide contents of the roots compared to the control. P 1500 + O 500 also significantly (*p* < 0.05) improved their extractum contents. Simultaneously, the ash of P 1500 + O 500 was significantly (*p* < 0.05) greater than that of P 1500 and O 1000, and slightly greater than that of P 1000 and O 500; their total saponin and polysaccharide contents were significantly (*p* < 0.05) greater than those of P 1000, P 1500, O 500, and O 1000, and their extractum content was slightly higher than that of P 1000, P 1500, O 500, and O 1000. These findings show that the medicinal quality of *P. heterophylla* roots could be increased by the combination of low-dosage pyraclostrobin and oligochitosan, and that its effects were better than those of high-dosage pyraclostrobin.

## 3. Discussion

Oligochitosan can be applied as an agricultural antibiotic in order to inhibit the growth of pathogens and as a biostimulant inducer in order to activate the resistance of plants to disease [27,28,31,32,33]. In addition, pyraclostrobin is a broad-spectrum and efficient strobilurin fungicide [16,17,18,19,20,21]. Wang et al. [35] found that oligochitosan combined with tebuconazole could effectively improve kiwifruit’s resistance to soft rot disease. Subsequently, we found that the ability of pyraclostrobin to control powdery mildew in *R. roxburghi* could be effectively promoted by chitosan [21]. Meanwhile, oligochitosan is produced by the degradation of chitosan, which is obtained from chitin deacetylation [27,28]. In this study, P 1500 + O 500 were able to control LSD well, with effects of 87.49%~85.75%; these values are significantly (*p* < 0.05) greater than those obtained for P 1000 and dramatically (*p* < 0.01) higher than those for P 1500, O 500, and O 1000. These results demonstrate that oligochitosan in combination with low-dosage pyraclostrobin could more effectively control LSD compared to high-dosage pyraclostrobin, and that this combination effectively enhances the effects of low-dosage pyraclostrobin against LSD. This synergistic effect of oligochitosan and low-dosage pyraclostrobin against LSD was possibly obtained from the systemic, protective, and therapeutic effects of pyraclostrobin on plant pathogens, as well as the ability of oligochitosan to induce resistance in plants and its antibacterial activity against plant pathogens.

Phenols and flavonoids can participate in the biosynthesis of lignin and improve the ligninization of the host cells; disease-related proteins are important indicators of induced resistance, and MDA can directly reflect the peroxidation level of the cell plasma membrane [36,37]. The harm caused by reactive oxygen species can be alleviated by SOD; PPO is actively involved in lignin biosynthesis; and PAL is involved in the synthesis of defense substances, including lignin and phytoalexins. In addition, the H_2_O_2_ in lignin biosynthesis can be catalytically decomposed by POD [37]. Oligochitosan can stimulate the gene expression of disease-resistant substances in plants, including chitinase, glucanase, phytoalexin, and PR protein [30,31,32]. Wang et al. [35] found that oligochitosan could notably enhance the resistance substances and enzyme activity of kiwifruit, and Zhang et al. [21] reported that pyraclostrobin could partly enhance the phenolics, flavonoids, soluble protein, and SOD activity of *R. roxburghii*. In this work, oligochitosan combined with pyraclostrobin clearly enhanced the effects of their lone application on the protein, phenol, and flavonoid contents and SOD, POD, PAL, and PPO activities of the leaves; this combination also enhanced the inhibiting effects on the MDA level. These findings emphasize that the joint application of oligochitosan and low-dosage pyraclostrobin is able to enhance the potential resistance of *P. heterophylla* to LSD; they further emphasize that this formula shows outstanding results regarding the management of LSD and that it is able to reduce the application of pyraclostrobin.

Whether *P. heterophylla* plants are free from pests and diseases and have good growth determines their medicinal quality and yield. Many studies have demonstrated that oligochitosan can be widely used as a plant growth enhancer [27,28]. For instance, oligochitosan can effectively promote the photosynthesis and fruit production of tomatoes [28]; increase the biomass and yield of tomatoes via the foliar spraying of oligochitosan [33]; enhance the resistance and growth of kiwifruit [35]; improve the color intensity and anthocyanin content of grapes [38]; and improve the fruit quality of strawberries [39]. Furthermore, some studies have found that oligochitosan’s parent, chitosan, also effectively promotes the photosynthesis, yield, and quality of *R. roxburghii*, *Actinidia deliciosa*, *P. heterophylla*, and *Pinellia ternata* [21,35,36,40]. The results show that the joint application of oligochitosan and low-dosage pyraclostrobin can more effectively enhance the chlorophyll, Pn, Gs, Tr, and Ci contents of *P. heterophylla* than pyraclostrobin or oligochitosan alone; promote the total plant length, leaf area, stem diameter, aboveground biomass, underground biomass, and total biomass of the plant; reliably enhance its roots’ fresh weight, dry weight, length, and diameter; and notably enhance its roots’ ash, total saponins, polysaccharide, and extractum contents. These beneficial results were related to the combined application of oligochitosan and low-dosage pyraclostrobin in managing LSD in *P. heterophylla* and enhancing its growth; this also emphasizes that oligochitosan is an available synergist of pyraclostrobin and that it can be used to enhance the root yield and medicinal quality of *P. heterophylla*.

In recent years, the Chinese government has paid increasing attention to reducing the application of pesticides and enhancing the development of complementary or alternative methods in the management of pests and diseases [41]. In this study, the formulaic application of oligochitosan and low-dosage pyraclostrobin could more effectively control LSD in *P. heterophylla*, improve its resistance, photosynthesis, growth, yield, and quality, and clearly reduce pyraclostrobin use. Meanwhile, the pyraclostrobin dosage in its formula with oligochitosan was very low (1500-time diluent); oligochitosan is natural and nontoxic, and it has a safe interval period of over 80 d, which is extremely long. Furthermore, oligochitosan is widely applied in food, the chemical and energy industries, environmental protection, medicine, and other fields, such as preservation films, skin care facial masks, beverages, etc. Accordingly, the hidden risks posed by this formula were nonexistent. Overall, this study highlights that 30% pyraclostrobin SC 1500-time + 5% oligochitosan AS 500-time diluent can be suggested for use as a novel and practicable combination for the control of LSD in *P. heterophylla*, and that it can decrease pyraclostrobin use. Nevertheless, the synergistic mechanism by which oligochitosan and pyraclostrobin control LSD’s pathogen should be further studied; this would better reveal the ability of this formula to control LSD in *P. heterophylla*.

## 4. Materials and Methods

### 4.1. Oligochitosan, Pyraclostrobin, and Instruments

Five percent of oligochitosan AS was provided by Kesheng Group Co. Ltd. (Yancheng, Jiangsu, China), its trade name is Xiansheng. Thirty percent pyraclostrobin SC was produced by Jinan Zhongke Green Biological Engineering Co. Ltd. (Jinan, Shandong, China), its trade name is Cuijian. The portable LI-6400XT photosynthesis measurement system was produced by LI-COR Inc., (Lincoln, NE, USA), and the UV-5800PC spectrophotometer was produced by Shanghai Yuan Analysis Instrument Co., Ltd. (Shanghai, China).

### 4.2. Field Herbal Orchard

*P. heterophylla* herbal garden with ‘Shitai 1’ of the planting cultivar was used for the field control experiment, its LSD in the previous year at the herbal garden was severe. *P. heterophylla* seed roots were planted by ridging, every plot area in the herbal garden was 3.0 m^2^ (6.0 m of ridge width, 0.5 m of ridge length, and 0.2 m of ridge between), and the planting density of seed roots was 30 kg per 666.7 m^2^ and the weight of each seed root was 0.10–0.12 g. Moreover, the climate and fertility information of the *P. heterophylla* herbal garden are displayed in Table 2.

### 4.3. Control Test

The completely randomized and leaf spraying methods were used for designing test plots and spraying fungicide diluent, respectively. Then, six treatments were executed for controlling LSD: 30% pyraclostrobin SC 1500-time + 5% oligochitosan AS 500-time diluent (P 1500 + O 500), 30% pyraclostrobin SC 1000-time diluent (P 1000), 30% pyraclostrobin SC 1500-time diluent (P 1500), 5% oligochitosan AS 500-time diluent (O 500), 5% oligochitosan AS 1000-time diluent (O 1000), and water (Control), as well as each treatment set three replicates. An electrostatic atomizer was applied for spraying diluent. The pyraclostrobin or oligochitosan diluent was sprayed on the plant aboveground parts on 28 March, 4 April, and 11 April, respectively, and the used liquid amount of diluent every time was 60 L per 666.7 m^2^, and its spray rate was 45 L h^−1^.

### 4.4. Methods

#### 4.4.1. Control Effects

The leaf disease index was counted on day 15 and day 30 after the last spraying, according to Zhang et al. [36], respectively. Thirty plants were randomly sampled from the five parts in each plot, and their total and diseased leaf numbers were investigated. The classification of diseased leaf level:

0 level: no diseased spot;

1 level: the diseased spot area was lower than 5% of the leaf area;

3 level: the diseased spot area was 6~10% of the leaf area;

5 level: the diseased spot area was 11~20% of the leaf area;

7 level: the diseased spot area was 21~50% of the leaf area;

9 level: the diseased spot area was higher than 51% of the leaf area.

Subsequently, the disease index and control effect were respectively calculated as Equations (1) and (2):

Disease index = 100 × ∑ (leaf number of each level × level value)/(highest level value × leaf total number)(1)

Control effect (%) = 100 × (1−disease index of treatment plot/disease index of control plot)(2)

#### 4.4.2. Resistance-Related Substance Content and Enzyme Activity

Thirty *P. heterophylla* leaves were randomly sampled from the five parts in each plot to investigate their resistance parameters 30 days after the last spraying. Thiobarbituric acid and Coomassie brilliant blue methods were applied for measuring malonaldehyde (MDA) and soluble protein, respectively. Moreover, the total phenol and flavonoid contents were determined by the gallic acid and rutin standard curve methods, respectively. Meanwhile, the nitrogen blue tetrazole, guaiacol, trans-cinnamic acid, and catechol methods were applied for checking the superoxide dismutase (SOD), peroxidase (POD), phenylalaninammo nialyase (PAL), and polyphenoloxidase (PPO) activities, respectively. The detailed detection steps for all the above resistance parameters are according to Zhang et al. [42,43].

#### 4.4.3. Photosynthetic Capability and Agronomic Trait Parameters

The health of *P. heterophylla* leaves was randomly sampled from the five parts in each plot at 30 d after the last spraying to measure their chlorophyll, photosynthetic rate (Pn), transpiration rate (Tr), intercellular carbon dioxide concentration (Ci), stomatal conductance (Gs), and water use efficiency (WUE), according to Chen et al. [40] and Zhang et al. [42]. Ten *P. heterophylla* plants in the five positions of each plot were randomly chosen for monitoring their growth parameters on Jun 18. The total plant length, leaf length, leaf width, and stem diameter of *P. heterophylla* were determined by a ruler, and its leaf area was calculated by the leaf area coefficient (0.666) method: leaf area = 0.666 × leaf width × leaf length. After drying at low temperatures, their total biomass, underground biomass, and above-ground biomass were weighed by an electronic balance.

#### 4.4.4. Tuberous Root Growth and Medicinal Quality

*P. heterophylla* tuberous roots were sampled and washed on 7 July to check their growth and medicinal quality. The diameter and length of 100 tuberous roots in every plot were surveyed by vernier scale, as well as their fresh weight and dry weight, which were determined by the gravimetric and oven-drying methods. Meanwhile, the ash, total saponins, polysaccharide, and extractum contents of tuberous roots were determined, and the detailed detection steps referred to Chinese Pharmacopoeia 2020 [44].

### 4.5. Statistical Methods

The Duncan’s test with one-way analysis of variance on SPSS 18.0 (SPSS Inc., Chicago, IL, USA) was applied for analyzing the significant differences in the data. All figures were drawn with Origin 10.0 (OriginLab, Northampton, MA, USA).

## 5. Conclusions

Overall, oligochitosan can be used as a synergist to promote the ability of pyraclostrobin to control leaf spot disease in *P. heterophylla*. Moreover, the formulaic application of oligochitosan and pyraclostrobin increased the phenol, flavonoid, and protein levels as well as the SOD, POD, PAL, and PPO activities of the leaves compared to their individual applications. Furthermore, their joint application obviously enhanced the effects of their individual application on the leaf photosynthesis, plant agronomic traits, root growth, and root quality of *P. heterophylla*. This study shows that oligochitosan combined with pyraclostrobin can be suggested as a practicable and alternative measure to be employed for the control of leaf spot disease in *P. heterophylla*.

## Figures and Tables

**Table 1:** The control roles of pyraclostrobin and oligochitosan on LSD.

Treatments	Applied Concentrations (g per 666.7 m^2^ Each Time)	15 d after Spraying		30 d after Spraying	
		Disease Index	Control Role (%)	Disease Index	Control Role (%)
P 1500 + O 500	12 g pyraclostrobin + 6 g oligochitosan	0.68 ± 0.14 ^fD^	87.49 ± 2.27 ^aA^	0.98 ± 0.19 ^eE^	85.75 ± 2.21 ^aA^
P 1000	18 g pyraclostrobin	1.17 ± 0.24 ^eD^	78.45 ± 4.01 ^bA^	1.63 ± 0.21 ^dD^	76.17 ± 3.14 ^bB^
P 1500	12 g pyraclostrobin	1.75 ± 0.25 ^dC^	67.74 ± 4.03 ^cB^	2.31 ± 0.16 ^cC^	66.15 ± 3.91 ^cC^
O 500	6 g oligochitosan	2.34 ± 0.17 ^cB^	56.84 ± 0.67 ^dC^	2.85 ± 0.17 ^bBC^	58.31 ± 3.55 ^dCD^
O 1000	3 g oligochitosan	2.75 ± 0.15 ^bB^	49.03 ± 5.62 ^eC^	3.23 ± 0.23 ^bB^	52.86 ± 1.98 ^dD^
Control	-	5.42 ± 0.33 ^aA^		6.85 ± 0.24 ^aA^	

Data indicate the average value ± standard deviation. Uppercase and lowercase letters show significant differences at 1% (*p* < 0.01) and 5% (*p* < 0.05) levels, respectively.

**Table 2:** The climate and fertility information of the herbal garden.

Soil Properties	Content	Soil Properties	Content (mg kg^−1^)
Temperature	15.7 °C	Available zinc	2.04
Sunshine	1188.7 h	Available phosphorus	56.65
Rainfall	1004.4 mm	Available potassium	129.72
Frostless season	325 days	Available iron	8.42
Average altitude	1119 m	Available manganese	18.25
pH	4.95	Exchangeable magnesium	288.68
Organic matter	36.04 g kg^−1^	Available boron	0.21
Exchangeable calcium	20.83 cmol kg^−1^	Alkali hydrolyzed nitrogen	114.24

## Data Availability

The datasets analyzed in the current study are available from the corresponding author upon reasonable request.
